# Cytoprotective Potential of *Annurca* Apple Polyphenols on Mercury-Induced Oxidative Stress in Human Erythrocytes

**DOI:** 10.3390/ijms26188826

**Published:** 2025-09-10

**Authors:** Pasquale Perrone, Claudia Moriello, Nicola Alessio, Caterina Manna, Stefania D’Angelo

**Affiliations:** 1 Department of Medical, Human Movement, and Well-Being Sciences (DiSMMeB), Parthenope University of Naples, 80133 Naples, Italy; 2 Department of Experimental Medicine, University of Campania “Luigi Vanvitelli”, 80138 Naples, Italy; claudia.moriello@unicampania.it (C.M.); nicola.alessio@unicampania.it (N.A.); 3 Department of Precision Medicine, School of Medicine, University of Campania “Luigi Vanvitelli”, 80138 Naples, Italy; caterina.manna@unicampania.it

**Keywords:** apple extracts, erythrocytes, glutathione, mercury, oxidative stress, reactive oxygen species

## Abstract

Mercury (Hg) exposure is a major environmental risk factor, closely linked to oxidative stress and cardiovascular disease. Red blood cells (RBC), due to their high oxygen exposure and lack of repair mechanisms, are particularly sensitive to oxidative injury and are key indicators of systemic redox imbalance. This study evaluates the protective effects of polyphenolic extracts from *Annurca* apple, specifically flesh and peel, from both ripe and unripe fruit, on HgCl_2_-exposed human RBCs. Key oxidative stress markers were measured, including ROS production, GSH levels, lipid peroxidation (TBARS), MetHb formation, SH group content, microvesicle (MV) generation, and morphological changes. Peel extracts, particularly those from ripe apples, consistently exhibited stronger antioxidant and cytoprotective effects than flesh extracts, effectively reversing Hg-induced oxidative damage and preserving RBC integrity. Notably, these extracts restored redox homeostasis and GSH levels, reduced ROS and TBARS accumulation, prevented MetHb formation, and mitigated MV release and morphological alterations. These protective effects appear to involve multifactorial mechanisms. These findings highlight the nutraceutical potential of *Annurca* apple extracts in counteracting heavy metal-induced oxidative stress and support their possible relevance for future studies aimed at health protection and waste valorization.

## 1. Introduction

*Malus pumila Miller* cv. *Annurca*, commonly known as the *Annurca* apple, is a traditional cultivar native to the Campania region of Southern Italy. It is registered as a Protected Geographical Indication (IGP) product and is increasingly recognized for its nutraceutical properties [1]. Characterized by a phytochemical profile particularly rich in polyphenols, including procyanidins, flavonols, and phenolic acids, the *Annurca* apple has demonstrated numerous in vitro and in vivo biological activities [2,3,4]. Recent studies have highlighted its antioxidant, hypocholesterolemic, and anti-inflammatory effects, as well as its ability to positively modulate gut microbiota [5,6,7,8,9,10,11]. However, to date, the potential protective effects of *Annurca* apple polyphenolic extracts against heavy metal-induced cellular toxicity have not been fully explored.

Among heavy metals with the greatest toxicological relevance, mercury (Hg) is considered one of the most hazardous to both human health and the environment [12]. In nature, Hg exists in three main chemical forms: elemental mercury (Hg^0^), inorganic mercury (Hg^+^ and Hg^2+^), and organic mercury, primarily in the form of methylmercury (MeHg) [13]. Elemental Hg occurs as vapor and is mainly released through anthropogenic activities such as mining, coal combustion, and waste incineration. Inorganic forms are typically encountered in industrial environments, whereas MeHg is primarily produced through bacterial methylation in aquatic sediments [14]. It is the most toxic and bioavailable form to humans, due to its tendency to bioaccumulate and biomagnify along the food chain, reaching high concentrations in fish and seafood [15].

Hg toxicity in humans most frequently derives from chronic low-level exposure, primarily through the dietary intake of MeHg-contaminated fish and seafood. This long-term accumulation represents the prevailing and most challenging toxicological condition, being closely associated with cardiovascular, neurological, and developmental disorders [16]. Nevertheless, acute exposure models using inorganic Hg salts, such as HgCl_2_, are widely employed in vitro to reproduce a rapid and reproducible oxidative insult. These acute paradigms, although not fully representative of chronic intoxication, provide a valuable experimental tool to dissect the cellular and molecular mechanisms of redox imbalance.

Once absorbed, Hg can cross biological membranes and distribute within tissues, where it exerts its toxic effects through various mechanisms, primarily by interacting with thiol groups in proteins and glutathione (GSH). These interactions lead to enzyme dysfunctions, alterations in membrane permeability, and redox imbalances [17]. One of the primary pathogenic mechanisms of Hg-induced cellular damage is the overproduction of reactive oxygen species (ROS), which results in oxidative stress [18,19]. Blood cells, particularly human erythrocytes (RBC), are especially vulnerable to Hg-induced oxidative damage due to their high oxygen exposure, abundance of hemoglobin, and lack of nuclear regenerative systems [20,21].

RBCs therefore represent an excellent cellular model for studying oxidative stress induced by toxic agents, as they provide a simple, reproducible, and well-characterized system for evaluating morphological, biochemical, and functional changes in response to oxidative stimuli [22]. In this context, the investigation of natural agents with potential protective activity against Hg-induced toxicity in RBCs is of particular interest, both for understanding cellular defense mechanisms and for showing new strategies for the prevention and mitigation of heavy metal–induced damage.

In the present study, the effect of polyphenolic extracts from *Annurca* apple was investigated for their potential to counteract Hg-induced alterations in human RBCs, to evaluate the efficacy of this functional food as a protective agent against metal-induced oxidative stress. We employed both flesh and peel extracts of *Annurca* apples, aiming not only to compare their bioactivity but also to explore a sustainable use of the entire fruit, including often-discarded parts such as the peel. Furthermore, we examined both mature and immature apples to evaluate whether the ripening stage influences the protective effects and to promote the valorization of suboptimal or non-commercial fruits, in line with sustainability principles.

In a previous study, we demonstrated that, through UHPLC-MS/MS analysis, a total of fourteen phenolic compounds (catechin, epicatechin, quercetin, quercetin hexoside, 4-hydroxybenzoic acid, protocatechuic acid, OH-tyrosol, vanillic acid, caffeic acid, ferulic acid, chlorogenic acid, rutin, syringic acid, p-coumaric acid) are present in extracts of the *Annurca* apple. The analysis also revealed differences in phenolic composition between the various parts of the *Annurca* apple (peel and flesh) and between different ripening stages (unripe and ripe). In unripe samples, key polyphenols such as epicatechin, catechin, chlorogenic acid, and quercetin hexoside were consistently detected, especially in the peel and flesh. In contrast, ripe samples showed the presence of compounds such as 4-hydroxy-3-benzoic acid and quercetin. Ripe flesh displayed the highest diversity of polyphenols, including both flavonoids and phenolic acids, whereas the ripe peel exhibited a comparatively reduced profile [23].

To study the potential protective effect, several biochemical and structural markers of oxidative stress and cellular damage were assessed, including intracellular ROS production, reduced GSH levels, methemoglobin (MetHb) formation, total sulfhydryl (-SH) group content, and lipid peroxidation measured as thiobarbituric acid reactive substances (TBARS). In addition to biochemical aspects, Hg-induced morphological changes in RBCs were also examined through microscopic observation, as well as the formation of extracellular microvesicles (MVs), a phenomenon closely associated with the activation of pro-apoptotic pathways and cellular membrane destabilization.

The results obtained provide a detailed and comprehensive overview of the potential cytoprotective effects of *Annurca* apple extracts, contributing to a better understanding of their role in modulating Hg-induced oxidative damage in human RBCs.

## 2. Results

### 2.1. RBC Viability

**Figure 1:**
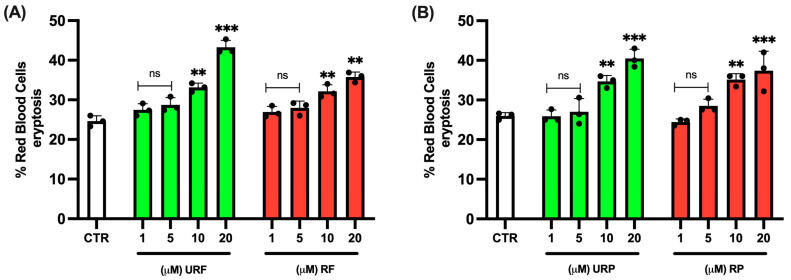
Effect of different *Annurca* apple extracts on RBC eryptosis. Cells were treated with increasing concentrations of (**A**) unripe flesh and ripe flesh (**B**) unripe peel and ripe peel. Data are the means ± SD (n = 3). Statistical analysis was performed with ANOVA followed by Tukey’s test. *** (*p* < 0.001) and ** (*p* < 0.01) show a significant difference from CTR. ns shows no significant difference from CTR.

To evaluate the potential cytotoxic effects of the extracts on RBC viability, we first performed an Annexin V assay (Figures S1 and S2). As shown in Figure 1A, both ripe (RF) and unripe (URF) apple flesh extracts did not induce significant levels of cell death at low concentrations (1 and 5 μM). However, a marked increase in eryptosis was observed at higher concentrations (10 and 20 μM). A similar trend was noted for peel extracts from both ripe (RP) and unripe (URP) apples as while the 1 and 5 μM concentrations were well tolerated, the 10 and 20 μM concentrations resulted in substantial toxicity.

Based on these findings, the higher concentrations (10 and 20 μM) were excluded from the next analyses to avoid confounding the interpretation of the data.

### 2.2. Total Sulfhydryl Group Content

The measurement of SH groups is a key indicator for evaluating the cellular redox state and the extent of oxidative stress. SH groups, present in proteins and small molecules such as GSH, are highly reactive towards ROS and other oxidizing agents. A decrease in –SH levels is generally associated with oxidative damage at the cellular level, leading to impairment of cellular function. Therefore, quantifying SH groups in RBCs represents a crucial parameter to monitor the toxic effects of oxidative agents, such as heavy metals, and to assess the protective potential of antioxidant compounds [24].

**Figure 2:**
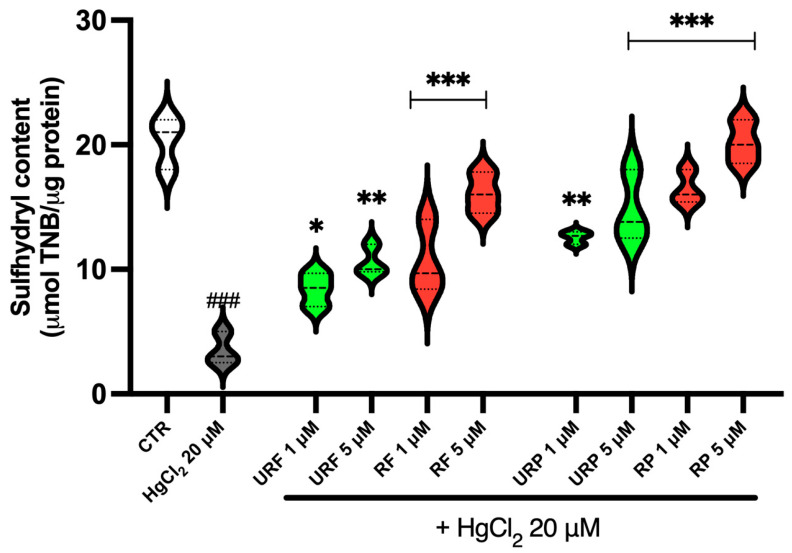
Effect of HgCl_2_ and different *Annurca* apple extract on RBC SH group content. Cells were treated with HgCl_2_ and increasing concentrations of URF, RF, URP and RP. Data are the means ± SD (n = 3). Statistical analysis was performed with one-way ANOVA followed by Tukey’s test. ### (*p* < 0.001) shows a significant difference from CTR. *** (*p* < 0.001), ** (*p* < 0.01) and * (*p* < 0.05) show a significant difference fromHgCl_2_ treatment.

The data presented in Figure 2 show that *Annurca* apple extracts can counteract the toxic effects of HgCl_2_ on SH group levels in RBCs. Specifically, a marked reduction in –SH groups is observed following treatment with the heavy metal, confirming the induction of oxidative stress. However, treatment with extracts from unripe and ripe apple flesh resulted in a concentration-dependent increase in –SH group levels. The most pronounced effect was observed with RF at a concentration of 5 μM.

Similarly, extracts from unripe and ripe apple peel also showed a dose-dependent increase in SH groups, with the strongest effect observed at 5 μM. Notably, the peel extracts demonstrated a stronger antioxidant effect compared to the flesh extracts, likely due to the higher concentration of phenolic compounds in the peel.

### 2.3. MetHb Levels

MetHb is an oxidized form of hemoglobin in which the heme iron is in the ferric (Fe^3+^) state and is unable to bind and transport oxygen. Intracellular accumulation of MetHb is a well-established marker of oxidative stress, as it results from exposure to oxidizing agents that disrupt the redox balance of cells. In RBCs, which lack nuclei and mitochondria, hemoglobin integrity is especially susceptible to oxidative damage. Therefore, the quantification of MetHb provides valuable information for assessing oxidative injury and evaluating the protective efficacy of antioxidant compounds [25].

**Figure 3:**
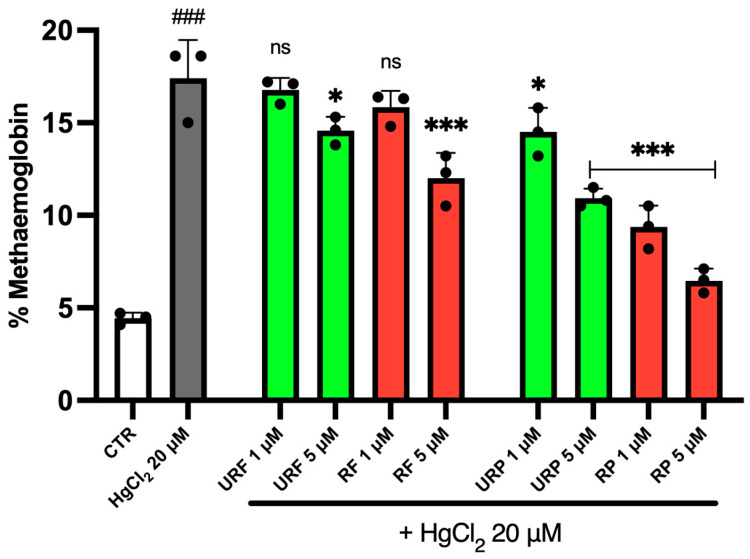
Effect of HgCl_2_ and different *Annurca* apple extract on RBC MetHb content. Cells were treated with HgCl_2_ and increasing concentrations of URF, RF, URP and RP. Data are the means ± SD (n = 3). Statistical analysis was performed with one-way ANOVA followed by Tukey’s test. ### (*p* < 0.001) shows a significant difference from CTR. *** (*p* < 0.001) and * (*p* < 0.05) indicate a significant difference fromHgCl_2_ treatment. ns shows no significant difference fromHgCl_2_.

Intracellular MetHb levels are reported in Figure 3. As shown, treatment with HgCl_2_ resulted in a significant increase in MetHb levels, more than a threefold rise, indicating pronounced oxidative stress. However, treatment with *Annurca* apple extracts partially reversed this effect.

Interestingly, for flesh extracts from both unripe (URF) and ripe (RF) apples, the protective effect was clear only at the highest concentration tested (5 μM). In contrast, peel extracts from unripe (URP) and ripe (RP) apples exerted a protective effect against metal-induced toxicity at all tested concentrations, in a dose-dependent manner. Among these, RP extracts proved the strongest effect, suggesting that bioactive compounds in ripe apple peel play a key role in counteracting HgCl_2_-induced oxidative stress.

### 2.4. TBARS Levels

Thiobarbituric Acid Reactive Substances (TBARS) are commonly measured as an indirect index of lipid peroxidation, a key process in oxidative damage. When ROS attack polyunsaturated fatty acids in cellular membranes, they start chain reactions that lead to the formation of lipid peroxides. One of the most studied end-products of this process is malondialdehyde (MDA), which reacts with thiobarbituric acid to form a colored complex measurable spectrophotometrically. Elevated TBARS levels thus reflect increased membrane lipid damage and are widely used as a reliable biomarker of oxidative stress, particularly in models involving exposure to toxic agents such as heavy metals [26].

**Figure 4:**
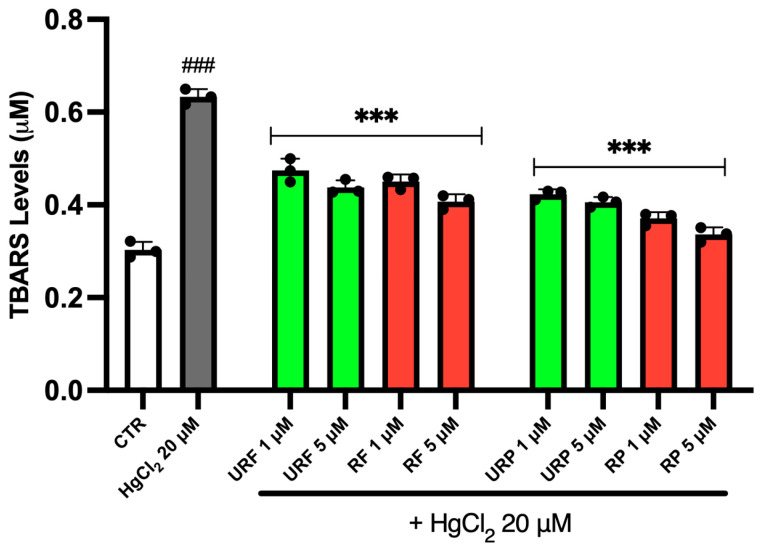
Effect of HgCl_2_ and different *Annurca* apple extract on RBC TBARS levels. Cells were treated with HgCl_2_ and increasing concentrations of URF, RF, URP and RP. Data are the means ± SD (n = 3). Statistical analysis was performed with one-way ANOVA followed by Tukey’s test. ### (*p* < 0.001) indicates a significant difference from CTR. *** (*p* < 0.001) indicates a significant difference fromHgCl_2_ treatment.

The variations in TBARS levels are shown in Figure 4. As illustrated, treatment with HgCl_2_ induced approximately a threefold increase in TBARS compared to physiological conditions, showing enhanced lipid peroxidation. Notably, *Annurca* apple extracts exerted a marked protective effect in this context. In all treatment groups, a substantial reduction in TBARS levels was observed following exposure to the apple extracts.

Interestingly, in this case, no major differences were observed among the URF, RF and URP extracts. However, the RP samples appeared to produce a slightly more pronounced effect, suggesting a higher antioxidant potential, possibly due to the greater concentration of phenolic compounds in the mature peel.

### 2.5. ROS Levels

ROS are chemically reactive molecules derived from oxygen that play a dual role in cellular physiology. At low levels, ROS function as signaling molecules, but under conditions of oxidative stress, such as exposure to toxicants or heavy metals, their accumulation can cause severe damage to cellular structures, including lipids, proteins, and DNA. Excessive ROS production disrupts redox homeostasis and is a hallmark of oxidative injury, making their quantification an essential tool for evaluating both the extent of oxidative stress and the efficacy of antioxidant interventions [27,28].

**Figure 5:**
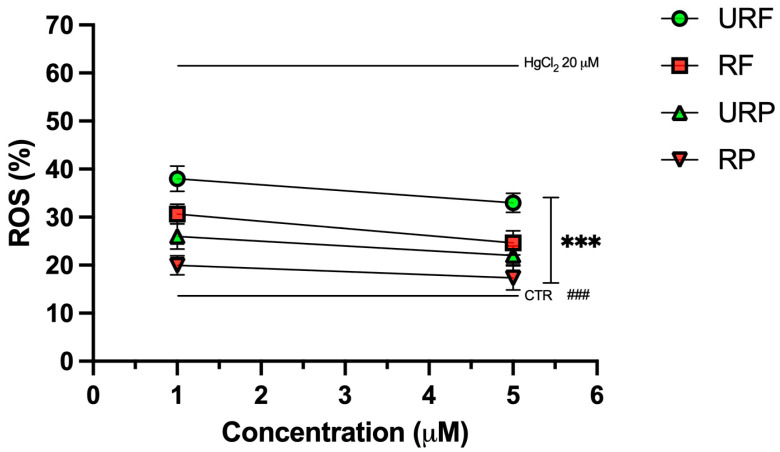
Effect of HgCl_2_ and different *Annurca* apple extract on RBC ROS generation. Cells were treated with HgCl_2_ and increasing concentrations of URF, RF, URP and RP. Data are the means ± SD (n = 3). Statistical analysis was performed with ANOVA followed by Tukey’s test. ### (*p* < 0.001) indicates a significant difference from CTR. *** (*p* < 0.001) indicates a significant difference fromHgCl_2_ treatment.

Intracellular ROS levels are presented in Figure 5. As shown, treatment with HgCl_2_ led to a marked increase in ROS production, approximately a threefold rise compared to physiological conditions confirming the pro-oxidant effect of the heavy metal. Notably, treatment with *Annurca* apple extracts significantly attenuated this effect.

For flesh extracts from unripe (URF) and ripe (RF) apples, a clear dose-dependent reduction in ROS levels was observed, with RF showing a more pronounced antioxidant effect than URF. A similar pattern was clear for peel extracts: both unripe (URP) and ripe (RP) samples demonstrated dose-dependent reductions in ROS, with RP showing the strongest effect overall.

Remarkably, treatment with RP at the highest tested concentration (5 μM) almost completely restored ROS levels to those observed under control conditions, suggesting a highly effective antioxidant response likely attributable to the rich phenolic content of the ripe peel.

### 2.6. GSH Content

GSH is one of the main endogenous antioxidants involved in cellular protection against oxidative stress. Its presence is essential for supporting membrane integrity and for the detoxification of free radicals and heavy metals. Therefore, measuring GSH levels represents a critical indicator of the cellular redox state and the efficacy of bioactive compounds in preventing or counteracting oxidative damage [29].

**Figure 6:**
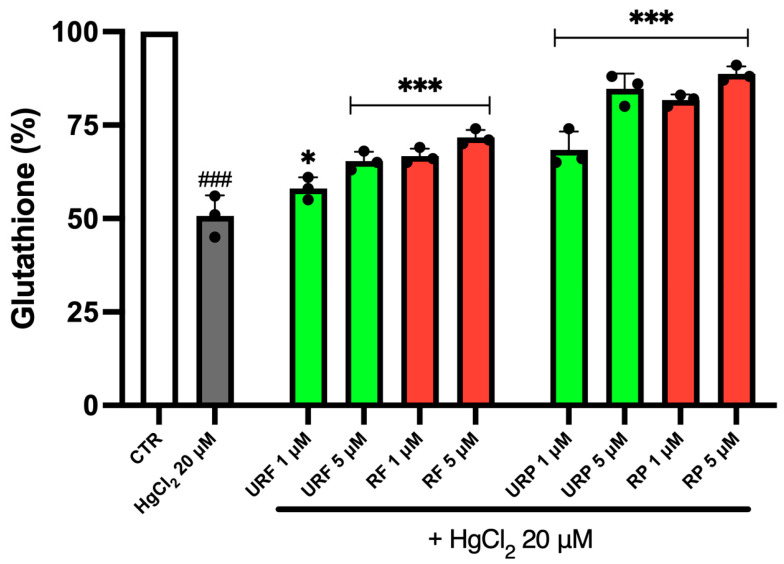
Effect of HgCl_2_ and different *Annurca* apple extract on GSH levels. Cells were treated with HgCl_2_ and increasing concentrations of URF, RF, URP and RP. Data are the means ± SD (n = 3). Statistical analysis was performed with one-way ANOVA followed by Tukey’s test. ### (*p* < 0.001) indicates a significant difference from CTR. *** (*p* < 0.001) and * (*p* < 0.05) indicate a significant difference fromHgCl_2_ treatment.

To evaluate the potential protective effect of *Annurca* apple extracts, GSH content was measured. As shown in Figure 6, treatment with toxic metal resulted in an approximately 50% decrease in GSH levels in RBCs. However, a substantial increase in GSH content was observed following treatment with URF and RF extracts, in a dose-dependent manner. Again, extracts obtained from ripe apples were more effective than those from unripe apples. Similar results were obtained with peel extracts, with a particularly strong effect observed for the RP sample, especially at 5 μM. Overall, peel extracts proved to be more effective than flesh extracts in restoring GSH levels.

### 2.7. MV Generation

**Figure 7:**
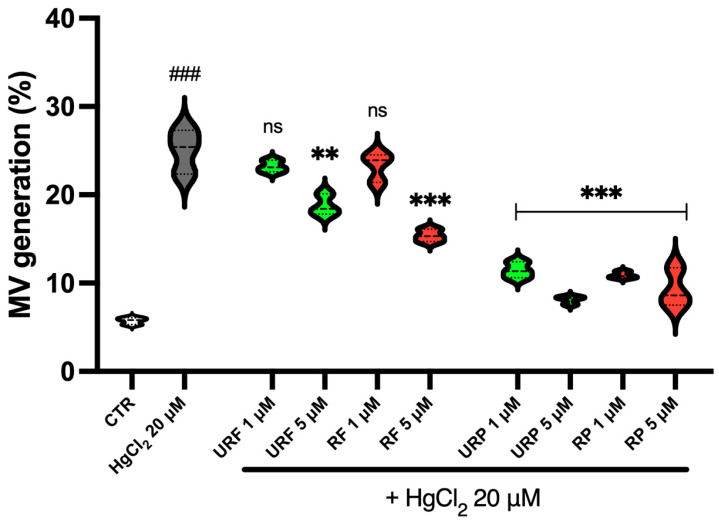
Effect of HgCl_2_ and different *Annurca* apple extract on MV generation. Cells were treated with HgCl_2_ and increasing concentrations of URF, RF, URP and RP. Data are the means ± SD (n = 3). Statistical analysis was performed with one-way ANOVA followed by Tukey’s test. ### (*p* < 0.001) indicates a significant difference from CTR. *** (*p* < 0.001) and ** (*p* < 0.01) indicate a significant difference from HgCl_2_ treatment. ns indicates no significant difference fromHgCl_2_ treatment.

Figure 7 shows data related to MV generation (Figures S3 and S4). As illustrated, treatment with the heavy metal resulted in a substantial increase in MV production, approximately five-fold compared to the control. In contrast, treatment with apple extracts significantly reduced MV generation.

Interestingly, flesh samples at low concentrations (1 μM), from both ripe and unripe apples, did not show any appreciable reduction in this process. However, flesh samples at higher concentrations (5 μM) showed a marked decrease in MV production.

Peel extracts, on the other hand, proved a strong protective effect regardless of concentration or ripening stage. Notably, in this case, the most effective sample appeared to be URP at 5 µM, which contrasts with the results observed previously.

### 2.8. Microscopic Analysis

**Figure 8:**
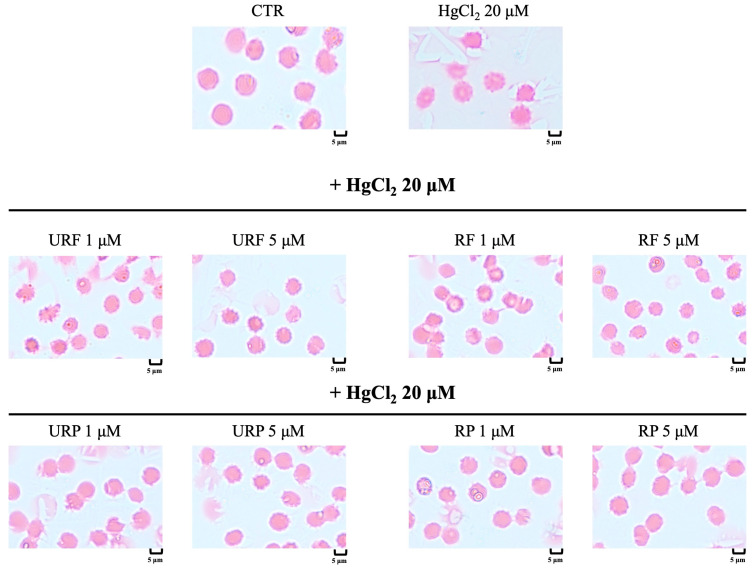
Effect of HgCl_2_ and different *Annurca* apple extract on RBC morphology. Cells were treated with HgCl_2_ and increasing concentrations of URF, RF, URP and RP. Optical microscope 100×.

Using Giemsa staining and optical microscopy, we evaluated whether Hg treatment induced morphological alterations in RBCs, as well as the potential protective effect of *Annurca* apple extracts. As shown in Figure 8, cells exposed to heavy metal showed clear morphological abnormalities, with the appearance of acanthocytes (RBC characterized by spiky membrane protrusions). Treatment with apple extracts appeared to reverse these alterations. However, it is noteworthy that low concentrations (1 μM) of URF and RF were not sufficient to fully prevent the formation of acanthocytes, as their numbers remained relatively high under these conditions. In contrast, higher concentrations (5 μM) of the same flesh extracts restored physiological biconcave morphology in RBCs. Interestingly, treatment with peel extracts, regardless of concentration or apple ripening stage, consistently restored RBCs to their normal biconcave shape, suggesting a strong protective effect.

## 3. Discussion

Oxidative stress is a pathophysiological condition characterized by excessive production of ROS in the face of insufficient endogenous antioxidant defenses, resulting in damage to lipids, proteins, and DNA [30]. This condition is closely associated with numerous chronic degenerative diseases, among which cardiovascular diseases are prominent [31]. In particular, oxidative stress is a common denominator in atherosclerosis, hypertension, cardiomyopathy, and heart failure, as ROS overproduction inactivates nitric oxide, promotes vascular inflammation and myocardial remodeling, ultimately leading to endothelial dysfunction and plaque instability [32,33].

Among the factors that most significantly amplify this vicious cycle is Hg: exposure to Hg, especially in its inorganic form, depletes GSH, oxidizes lipoproteins, and enhances platelet aggregation, with documented increases in hypertension, arrhythmias, and post-infarction mortality [34]. RBCs serve as a privileged indicator of these alterations: lacking a nucleus, continuously exposed to high O_2_ pressures, and rich in thiol groups, they are particularly susceptible to lipid peroxidation, heme oxidation to MetHb, and GSH depletion. These events lead to loss of deformability, release of pro-thrombotic MV, and abnormal adhesion to the endothelium [35].

Therefore, preventing or mitigating oxidative stress is a strategic goal in reducing cardiovascular risk, and the identification of bioactive compounds capable of counteracting these mechanisms is of high scientific and clinical relevance.

The data presented here provide experimental evidence of the effectiveness of polyphenolic compounds from *Annurca* apple in preventing metabolic and morphological damage to human RBCs exposed in vitro to HgCl_2_. It is important to note that higher concentrations of *Annurca* apple extracts (>5 μM) induced significant cytotoxicity in human RBCs, as demonstrated by the initial viability tests. For this reason, subsequent experiments focused on the 1–5 μM range, balancing antioxidant efficacy and cellular tolerance. Defining the precise therapeutic window for these extracts is crucial, as excessive doses can induce adverse effects. Future studies should further refine the dose–response relationship and evaluate safety margins in different biological models to ensure optimal nutraceutical application.

The results suggest that these extracts do not merely scavenge ROS but exert a deeper influence on intracellular redox homeostasis. In particular, the near-complete restoration of redox balance, even under potentially irreversible Hg-induced stress, indicates that *Annurca* apple polyphenols not only counter direct oxidation but also actively enhance endogenous detoxification systems. Since mature human erythrocytes are anucleate and lack transcriptional capacity, the observed effects are unlikely to involve direct activation of transcription factors or epigenetic mechanisms in RBCs. Instead, they may rely on chemical interactions (e.g., nucleophilic scavenging of Hg^2+^, regeneration of oxidized thiols) and reinforcement of existing redox buffers. Nevertheless, it is worth noting that polyphenols such as those from *Annurca* apple have been shown in nucleated cells to activate upstream antioxidant pathways (e.g., Nrf2 signaling) and to modulate epigenetic mechanisms [36,37].

The recovery of total SH groups and reduced GSH, both significantly compromised by Hg exposure, supports a mechanism involving the preservation or stimulation of endogenous antioxidant defenses. In particular, the dose-dependent increase in thiol groups suggests that apple polyphenols act both as direct nucleophiles toward Hg^2+^ and as regenerators of oxidized cysteine residues [38]. This aligns with the behavior of other plant-derived polyphenols known to activate antioxidant signaling pathways, enhancing the expression of detoxifying enzymes [39].

Reduced GSH is a major determinant of intracellular antioxidant capacity, playing a critical role in redox balance maintenance, heavy metal detoxification, and prevention of protein oxidation [40]. In RBCs, which lack protein synthesis and structural repair mechanisms, GSH is essential not only for ROS neutralization but also for regenerating oxidized thiol groups and preserving hemoglobin function [41]. Its depletion, triggered by Hg, is a pivotal event in the cascade leading to membrane rigidity, loss of biconcave morphology, and MV release [20]. The observation that *Annurca* apple extracts not only prevent GSH loss but promote its recovery in a dose-dependent manner has important pathophysiological implications.

Beyond enhancing synthesis, polyphenols may also protect GSH from oxidation or Hg conjugation. Certain phenolic compounds such as catechins and procyanidins, abundant in *Annurca* apple, exhibit chelating activity toward bivalent cations, thus reducing the chemical pressure on the GSH pool and preventing excessive depletion [42]. This preservation of GSH availability results in immediate protection against oxidative damage and enhances cellular resilience to later stress, laying the groundwork for a sustained protective effect.

Interestingly, proper GSH levels in the RBCs compartment also influence systemic vascular biology. Subclinical hemolysis and GSH instability in RBCs have been shown to release free hemoglobin and trigger endothelial inflammatory responses via secondary ROS and reduced nitric oxide bioavailability [43]. Therefore, the GSH-protective action of *Annurca* extracts may have broader implications, contributing to systemic vascular homeostasis beyond individual cellular protection.

Importantly, the protective effects observed extend beyond ROS scavenging to include the prevention of lipid peroxidation, as shown by reduced TBARS levels. Controlling TBARS is central to the protective activity of *Annurca* extracts since these compounds, derived from the reaction between malondialdehyde (MDA) and thiobarbituric acid, reflect the extent of membrane lipid peroxidation. The RBC membrane, rich in polyunsaturated fatty acids, is particularly vulnerable to radical attack, especially under Hg-induced redox stress, which promotes the formation of unstable lipid hydroperoxides [44].

The increase in TBARS after Hg exposure highlights extensive structural damage to the membrane, compromising integrity and increasing rigidity, which in turn impairs RBC deformability. *Annurca* apple extracts significantly reduced TBARS levels, suggesting an effective interruption of lipid peroxidation chain reactions. This may arise from direct scavenging of peroxyl and hydroxyl radicals, as well as from physical-chemical stabilization of membranes via hydrophobic interactions between polyphenols and lipid domains [24]. Notably, some flavonoids, such as procyanidins from *Annurca* peel, can insert into the lipid bilayer, preventing lateral diffusion of radicals and limiting the abnormal membrane fluidity typically seen under oxidative stress [45].

Moreover, lipid peroxidation is self-amplifying: secondary products such as MDA and 4-HNE (4-hydroxynonenal) are cytotoxic and pro-inflammatory, reacting with nucleophilic groups on proteins and DNA, thereby perpetuating systemic inflammation [46]. In this light, the TBARS-lowering effect of *Annurca* extracts not only confers structural protection but also interrupts a vicious pro-oxidative and pro-inflammatory cycle. This has clinical relevance in cardiovascular disease, where lipid peroxidation contributes directly to endothelial dysfunction, nitric oxide depletion, and leukocyte adhesion [47].

The ability of *Annurca* extracts to reduce TBARS levels even at low concentrations reinforces their potential pharmacological relevance, suggesting utility not only in acute toxic exposure but also in chronic conditions marked by persistent oxidative stress, such as diabetes, hypertension, or metabolic syndrome.

Simultaneous reduction in intracellular MetHb further confirms that the extracts preserve both membrane structure and hemoglobin function, critical in organelle-free cells lacking regenerative capacity. Reducing MetHb indicates that the extracts favor retention of the ferrous heme state, likely via reductive equivalents and chelation of pro-oxidant metals. Hemoglobin integrity is not only essential for oxygen transport but also for preventing immune activation: heme oxidation may generate immunogenic neoepitopes and promote DAMP (damage-associated molecular pattern) release, thus amplifying vascular inflammation [48]. Therefore, the inhibition of this cascade by polyphenols represents an additional protective mechanism with both redox and immunomodulatory implications. The consistent dose-dependent effects across all measured parameters further support the biological relevance of these findings, indicating genuine pharmacodynamic activity.

Another key finding concerns the alteration of the physiological morphology of RBCs, in particular the transformation into acanthocytes accompanied by the release of MV following prolonged exposure to low doses of HgCl_2_. These morphological alterations are not merely secondary to oxidative stress but reflect profound and functionally significant changes in RBC membrane biomechanics [49]. RBCs are highly specialized cells whose primary function, oxygen and carbon dioxide transport, critically depends on their characteristic biconcave shape, which provides a high surface-to-volume ratio and remarkable deformability. This morphology is essential for RBCs to navigate through narrow capillaries without undergoing lysis or losing functional capacity [50].

The shift toward acanthocytic forms, observed after HgCl_2_ exposure, leads to significant loss of cellular flexibility, increased membrane rigidity, and reduced deformability. This not only impairs gas transport efficiency but can also promote intravascular hemolysis or premature clearance of RBCs by the reticuloendothelial system, with potentially important hematological and microcirculatory consequences [51]. These morphological alterations often result from oxidative damage to key membrane components, such as thiol-containing structural proteins (e.g., spectrin and ankyrin), phospholipid peroxidation, and hemoglobin oxidation to MetHb, events that destabilize the cytoskeleton and trigger MV formation [52].

In this context, treatment with polyphenolic extracts from *Annurca* apple demonstrated a clear capacity to preserve RBC morphological integrity, preventing the transition to acanthocytes and significantly reducing MV generation. This protective effect is particularly relevant given that MV formation is not only a marker of oxidative stress and membrane damage, but also a mechanism of intercellular communication associated with pro-inflammatory and pro-thrombotic signaling, with direct implications for cardiovascular diseases [53]. Therefore, the ability of *Annurca* polyphenols to support the physiological biconcave shape of RBCs reflects not only their antioxidant and cytoprotective activity, but also a tangible preservation of hemorheological and hemodynamic function. This reinforces the hypothesis that polyphenols from *Annurca* apple can act as bioactive agents not only in controlling oxidative stress, but also in supporting efficient blood flow and protecting microcirculation, central aspects in the prevention of vascular complications associated with toxicity and chronic systemic stress [54]. However, these results derive exclusively from an in vitro RBC model and claims regarding cardiovascular protection must be considered preliminary. Further in vivo studies are required to confirm whether such protective effects on RBC morphology translate into systemic cardiovascular benefits.

From a pathophysiological perspective, protecting the structure and function of RBCs has significant implications for blood viscosity, laminar flow, and capillary perfusion. In various disease states, such as diabetes, hypertension, or chronic metal intoxication, RBC compartment dysfunction is often one of the earliest measurable alterations, and its correction represents an emerging therapeutic strategy in the field of cardio hemorheology [55,56].

These findings align well with growing evidence on the cardioprotective role of polyphenols. Meta-analyses and clinical intervention studies have shown that diets rich in flavonoids lower blood pressure, improve endothelial function, and reduce LDL oxidation [57]. Experimental studies in models of heart failure also support the ability of polyphenolic compounds, such as quercetin and resveratrol, to attenuate hypertrophy and improve ejection fraction via modulation of protective pathways like RISK (Reperfusion Injury Salvage Kinase) and SAFE (Survivor Activating Factor Enhancement) [58,59]. Our results extend this framework by demonstrating that *Annurca* polyphenols, in addition to their known vascular effects, can protect the RBC compartment from heavy metal-induced damage, an important finding given that RBC dysfunction precedes and fuels the thromboinflammatory cascade linking oxidative stress to cardiovascular pathology. Nonetheless, the absence of pharmacokinetic data on *Annurca* polyphenols, including absorption, distribution, metabolism, and achievable plasma concentrations, limits the extent to which nutraceutical applicability can currently be claimed.

A particularly relevant finding from our data is the superior efficacy of peel-derived extracts compared with those obtained from the flesh, likely due to the higher concentration of polyphenolic compounds in the outer layer of the fruit [23]. The peel of ripe *Annurca* apples showed the highest antioxidant and cytoprotective activity, both biochemically and morphologically. From a translational standpoint, the high efficacy of peel fractions suggests potential valorization of agro-industrial waste within a circular economy model, while ensuring standardization through the IGP certification of *Annurca* apple. The effective concentrations tested (1–5 μM) correspond to a daily intake of 300–600 mg of extract, a dose already attainable through nutraceutical formulations and compatible with plasma levels observed in dietary intervention studies. This supports the development of *Annurca*-based preparations for individuals exposed to Hg (e.g., habitual consumers of contaminated seafood) or patients with conditions marked by pro-oxidative risk.

An important methodological aspect concerns the experimental conditions used, particularly the concentrations of HgCl_2_ and *Annurca* extracts. In our model, RBCs were exposed to 20 µM HgCl_2_, a dose higher than that typically found in vivo, but widely employed in vitro to induce reproducible oxidative stress and allow the evaluation of protective effects. Likewise, *Annurca* extracts were tested at 1–5 µM, a range chosen to avoid cytotoxicity while ensuring measurable antioxidant activity. Although these levels do not perfectly reflect dietary exposure, comparable concentrations can be achieved through standardized nutraceutical formulations of *Annurca* polyphenols. Therefore, the adopted conditions should be regarded as a useful experimental model to uncover protective mechanisms, rather than a direct reproduction of real-life exposure

In conclusion, the multifactorial protection exerted by *Annurca* apple extracts on Hg-stressed RBCs is a novel contribution to the field of polyphenol-based cardioprotection. By preserving RBC integrity, these compounds may limit the upstream activation of pro-thrombotic and pro-inflammatory circuits that drive cardiovascular disease. Thanks to the availability of certified raw material and the sustainability of the extraction process, *Annurca* polyphenolic fractions emerge as promising candidates for future nutraceutical or pharmaceutical interventions aimed at preventing cardiovascular disorders linked to oxidative stress and heavy metal toxicity.

Although this study provides in-depth knowledge of the antioxidant and cytoprotective potential of *Annurca* apple extracts in vitro, it is limited by the absence of in vivo or systemic data. Human RBCs are a valuable model for studying oxidative damage; however, the complexity of cardiovascular physiology and heavy metal metabolism in whole organisms cannot be fully replicated in vitro. Therefore, extrapolation of the observed cardiovascular health benefits should be done with caution. Controlled preclinical animal studies and clinical trials are essential next steps to validate the efficacy, bioavailability, safety, and therapeutic potential of *Annurca* polyphenols in reducing cardiovascular risk associated with Hg exposure.

## 4. Materials and Methods

### 4.1. Chemicals and Solutions

DCFH-DA (2′,7′-dichlorodihydrofluorescein diacetate), DTNB (5,5-dithiobis (2-nitrobenzoic acid) or Ellman’s reagent), PBS (phosphate-buffered saline), EDTA, TCA, TBA and HgCl_2_ were purchased from Sigma Chemical Co. (St. Louis, MO, USA). The AnnexinV-fluorescein isothiocyanate (V-FITC) apoptosis detection kit (556547, BD Pharmigen, Franklin Lakes, NJ, USA) and anti-glycophorin A (FITC) antibody were purchased from www.antibodies-online.com (accessed on 25 May 2025). Giemsa stain was purchased from Dasit group.

### 4.2. Fruit Collection

*Annurca* apples (*Malus pumila* Mill. cv. *Annurca*) were harvested in 2024 from an orchard located in Giugliano in Campania (Naples, Italy). The fruits were harvested in September in the pre-climacteric phase, characterized by green skin and incomplete ripeness. Some of these unripe apples were at once processed for analytical purposes. The remaining fruits underwent the traditional post-harvest reddening process in “melai” (apple drying rooms), where the apples were exposed to natural sunlight for about a month (October). After the reddening phase, samples of ripe fruits were collected and processed for comparative analysis.

### 4.3. Polyphenol Extraction

Forty grams of *Annurca* apple sample were homogenized for 5 min by a Tefal rondo 500 homogenizer using 40 mL of 80% methanol and 20% water plus 0.18 N HCl (15 mL 12 N of HCl/L). After centrifugation (18,000× *g* for 25 min), the slurry was dried under vacuum by using the Univapor Concentrator Centrifuge, model Univapo 100 H (Uni Equip). The dried extracts were dissolved in 10 mL of PBS and frozen at −80 °C until use (Table 1).

The total polyphenolic content of apple extracts was estimated using the Folin–Ciocalteu phenolic reagent. The extracts (100 μL) were mixed with Folin–Ciocalteu phenolic reagent (0.5 mL), deionized water (0.9 mL), and Na_2_CO_3_ (7.5% *w*/*v*, 4 mL). The absorbance at 765 nm was measured 2 h after incubation at room temperature using a Cary ultraviolet-visible spectrophotometer. The measurement was compared to a standard curve of prepared catechin solutions and expressed in milligrams of catechin (CAE) equivalent per 100 g FW (fresh weight) of apple.

### 4.4. Preparation of RBC and Treatment with HgCl_2_ and Annurca Apple Extracts

Whole blood was obtained with informed consent from healthy volunteers at the University of Campania “Luigi Vanvitelli” (Naples, Italy). It was collected in heparinized tubes and centrifuged at 2000× *g* for 10 min at 4 °C. Subsequently, the buffy coat was removed from the samples, and the RBC fraction was washed twice with isotonic saline (0.9% NaCl) and resuspended in Krebs’ solution containing (mM) NaCl 125, KCl 4, MgSO_4_ 1, Hepes 32, CaCl_2_ 1, glucose 5; pH 7.4 to obtain different hematocrits for different experimental purposes. RBCs were incubated at 37 °C for 4 h with HgCl_2_ (20 μM) and increasing concentrations of the different *Annurca* apple extracts (1–5 μM).

### 4.5. Detection of Apoptotic RBC

After incubation under the respective experimental conditions, RBCs (1 × 10^6^ per condition) were resuspended in 600 μL of 1 × binding buffer and incubated in the dark for 15 min at room temperature with 5 μL of Annexin V Apoptosis Detection Kit. Fluorescence assessment was performed using FACS Calibur flow cytometer and data were analyzed with FlowJo v10 software (https://www.flowjo.com/solutions/flowjo, accessed on 25 May 2025).

### 4.6. Total Sulfhydryl Group Content

The measurement of total -SH groups was performed according to Perrone et al. [24]. RBCs (35% hematocrit) were centrifuged (1200× *g*, 5 min), and a 100 μL sample was hemolyzed in 1 mL of distilled water.

A 50 μL aliquot of the hemolysate was added to 1 mL of phosphate-buffered saline (PBS, pH 7.4) having 1 mM EDTA. The reaction was started by adding 30 μL of 10 mM 5,5′-dithiobis-(2-nitrobenzoic acid) (DTNB), and samples were incubated for 30 min at 25 °C in the dark. Control samples without cell lysate or DTNB were processed in parallel.

After incubation, absorbance was measured at 412 nm using UV-3100PC spectrophotometer. The concentration of 3-thio-2-nitrobenzoic acid (TNB) was determined after subtracting the absorbance of the blank (samples containing only DTNB). Data were normalized to total protein content and results are expressed as μM TNB/mg protein.

### 4.7. Determination of MetHb Levels

MetHb levels were determined as described by Naoum et al. [60], with minor modifications. The assay is based on the differential absorbance of MetHb and oxyhemoglobin (Hb) at 630 nm and 540 nm, respectively, measured by spectrophotometry.

The following incubation samples were centrifuged (2000× *g*, 5 min, 25 °C). A 25 μL aliquot of RBCs at 40% hematocrit was lysed in 1975 μL of hypotonic buffer (2.5 mM NaH_2_PO_4_, pH 7.4, at 4 °C).

The lysates were then centrifuged at 13,000× *g* for 15 min at 4 °C to remove cell membranes. The absorbance of the supernatant was measured using UV-3100PC spectrophotometer.

MetHb levels were expressed as a percentage of total hemoglobin using the following Equation:

% MetHb = (OD_630_/OD_540_) × 100,

where OD represents optical density.

### 4.8. TBARS Level Measurement

TBARS levels were measured as described by Mendanha et al. [61], with minor modifications. TBARS result from the reaction between thiobarbituric acid (TBA) and malondialdehyde (MDA), a final product of lipid peroxidation.

RBCs were suspended at 20% hematocrit and after incubation, samples were centrifuged (1200× *g*, 5 min) and resuspended in isotonic solution.

A 1.5 mL aliquot of RBC suspension was treated with 10% (*w*/*v*) trichloroacetic acid (TCA) and centrifuged (3000× *g*, 10 min). Then, 1 mL of 1% TBA solution (prepared in hot distilled water) was added to the supernatant, and the mixture was incubated at 95 °C for 30 min.

TBARS levels were quantified spectrophotometrically by subtracting 20% of the absorbance at 453 nm from the absorbance at 532 nm [UV-3100PC spectrophotometer (Avantor, Inc., Radnor, PA, USA)]. Results are expressed as μM TBARS, using a molar extinction coefficient of 1.56 × 10^5^ M^−1^·cm^−1^.

In our study, TBARS was used as a general indicator of oxidative damage, and the results should be interpreted accordingly.

### 4.9. ROS Determination

ROS generation was assessed using the DCF assay, as described by Notariale et al. [20]. Briefly, 250 μL of intact RBCs at 10% hematocrit were incubated with 10 μM 2′,7′-dichlorodihydrofluorescein diacetate (DCFH-DA) for 15 min at 37 °C.

After incubation, cells were centrifuged at 1200× *g* for 5 min at room temperature, the supernatant was discarded, and hematocrit was re-adjusted to 10% using Krebs solution. RBCs were then treated in the dark for 4 h.

Following treatment, 20 μL of RBC suspension were diluted in 2 mL of distilled water, and the fluorescence intensity of the oxidized product dichlorofluorescein (DCF) was measured at 502 nm excitation and emission 520 nm.

ROS levels were expressed as fluorescence intensity per mg of hemoglobin. Fluorescence measurements were performed using a UV-3100PC spectrophotometer.

A limitation of this study is that DCFH-DA fluorescence measurements in RBCs may be affected by hemoglobin absorbance and cellular autofluorescence. Since no specific correction was applied in our experimental setup, the reported fluorescence values should be interpreted as relative rather than absolute measures of ROS production.

### 4.10. GSH Assay

Intracellular GSH content was determined via reaction with DTNB. Following incubation, 0.25 mL of RBC suspension was centrifuged at 800× *g* for 5 min. After removal of the supernatant, RBCs were lysed by adding 0.6 mL of cold distilled water.

Proteins were precipitated by adding 0.6 mL of a cold metaphosphoric acid solution (prepared with 1.67 g metaphosphoric acid, 0.2 g EDTA, and 30 g NaCl in 100 mL of distilled water). The samples were incubated at 4 °C for 5 min, then centrifuged at 18,000× *g* for 10 min to remove the precipitated proteins.

A 0.45 mL aliquot of the resulting supernatant was mixed with an equal volume of 0.3 M Na_2_HPO_4_. To this mixture, 0.1 mL of DTNB solution (20 mg DTNB dissolved in 100 mL of water having 1% sodium citrate) was added. After 10 min of incubation at room temperature, absorbance was measured at 412 nm using UV-3100PC spectrophotometer.

### 4.11. Quantification of MV

The MV obtained after treatment with HgCl_2_ were counted by flow cytometry. In brief, centrifugation at 2000× *g* for 20 min at 4 °C was performed to remove cells and larger cell fragments. Next, the supernatant was centrifuged at 16,000× *g* for 60 min at 4 °C to isolate the MV. 95 μL of samples were mixed with 5 μL of anti-human glycophorin A FITC antibody and incubated for 20 min at room temperature on a roller. MV were quantified by flow cytometry, using FACS Calibur flow cytometer (Becton, Dickinson and Company, Franklin Lakes, NJ, USA). Data are expressed as values normalized to the control.

### 4.12. Microscopic Analysis

RBCs were stained using a standard Giemsa staining protocol to assess morphology and potential structural alterations. In brief, after the respective treatment conditions, the RBC suspensions were first washed three times with phosphate-buffered saline (PBS, pH 7.4) by centrifugation at 1500× *g* for 5 min at room temperature. A drop of the washed RBC suspension was placed on a clean microscope slide and spread evenly to obtain a monolayer. The smears were air-dried completely at room temperature. The dried smears were fixed in absolute methanol for 5 min and allowed to air dry. The slides were then incubated with freshly prepared 10% (*v*/*v*) Giemsa solution for 45–60 min at room temperature. After staining, the slides were gently rinsed with distilled water and left to air dry in an upright position. Once dry, the stained slides were examined under an optical microscope (100× oil immersion aim) for morphological analysis. Representative images were acquired using a digital camera system connected to the microscope.

### 4.13. Statistical Analyses

Data evaluations were expressed as means ± S.D. of 3 independent experiments performed in triplicate with RBCs from different donors. The significance of differences was determined by one-way ANOVA followed by a post Tukey’s multiple comparisons test. GraphPad Prism 10 was utilized for statistical analysis.

## 5. Conclusions

This study provides new evidence of the multifactorial cytoprotective effects exerted by polyphenolic extracts derived from the *Annurca* apple against Hg-induced oxidative stress in human RBCs. Our results demonstrate that, in particular, extracts from the peel of ripe apples restore redox homeostasis, attenuate markers of oxidative damage, and preserve cell morphology. These findings provide valuable insights for the emerging field of nutraceutical interventions targeting heavy metal detoxification and cardiovascular risk reduction.

However, challenges remain. The therapeutic window of the extracts requires further definition, especially regarding toxicity at higher concentrations. Current results suggest that the protective action of *Annurca* polyphenolic extracts extends beyond the simple removal of ROS. The observed restoration of GSH levels and total sulfhydryl groups indicates the activation of endogenous antioxidant defenses. Since mature RBCs are anucleate and unable to induce transcriptional responses, the activation of the Nrf2 transcription factor should be considered only as a speculative mechanism relevant to nucleated cells or in vivo models. Nevertheless, this pathway, which regulates the expression of antioxidant enzymes (HO-1, NQO1, GCLC), is supported by recent evidence of epigenetic modulation by procyanidins present in the peel. Furthermore, the metal-chelating properties of some polyphenols may directly reduce the bioavailability of Hg, thereby limiting redox cycling and cellular damage. Modulation of redox-sensitive enzymes and signaling pathways likely contributes further to multifaceted cytoprotection. Future mechanistic studies employing molecular and genetic approaches are needed to delineate these pathways and isolate the contribution of individual polyphenolic compounds.

Moreover, given the in vitro nature of this work, in vivo and clinical studies are essential to confirm the cardiovascular benefits and safety profile of *Annurca* polyphenol preparations. Future research should focus on analyzing the contribution of individual polyphenols, validating molecular mechanisms, and assessing efficacy in preclinical models. These steps are crucial for translating our promising results into effective strategies to reduce oxidative stress-related cardiovascular diseases associated with environmental heavy metal exposure.

## Figures and Tables

**Table 1:** Main polyphenolic compounds present in different fruit tissues (flesh, peel) and stages of ripeness (unripe vs. ripe) [23].

*Annurca* Apple Part	Polyphenolic Compounds
URF	Protocatecuic acid; tyrosol; caffeic acid; ferulic acid; epicatechin; catechin; clorogenic acid; quercetin hexoside; rutina.
RF	Protocatecuic acid; caffeic acid; ferulic acid; epicatechin; catechin; clorogenic acid; quercetin; quercetin hexoside; rutina.
URP	Caffeic acid; ferulic acid; epicatechin; catechin; clorogenic acid; quercetin hexoside; rutina.
RP	Protocatecuic acid; ferulic acid; epicatechin; clorogenic acid; quercetin hexoside; rutina.

## Data Availability

The data presented in this study are available on request from the corresponding author due to privacy.

## Supplementary Materials

The following supporting in-formation can be downloaded at: https://www.mdpi.com/article/10.3390/ijms26188826/s1.

## Abbreviations

The following abbreviations are used in this manuscript:

RBCs	Erythrocytes
GSH	Glutathione
Hg	Mercury
MetHb	Methemoglobin
MeHg	Methylmercury
MV	Microvesicles
ROS	Reactive oxygen species
SH	Sulfhydryl group
TBARS	Thiobarbituric acid reactive substances
