# Supplementary material for: Cytoprotective Potential of Annurca Apple Polyphenols on Mercury-Induced Oxidative Stress in Human Erythrocytes
Source: Int J Mol Sci. 2025 Sep 10;26(18):8826. doi: 10.3390/ijms26188826 (PMC12469998; doi:10.3390/ijms26188826)
Supplement: Supplementary file 1 [file ijms-26-08826-s001.zip › ijms-3825597-supplementary.pdf]

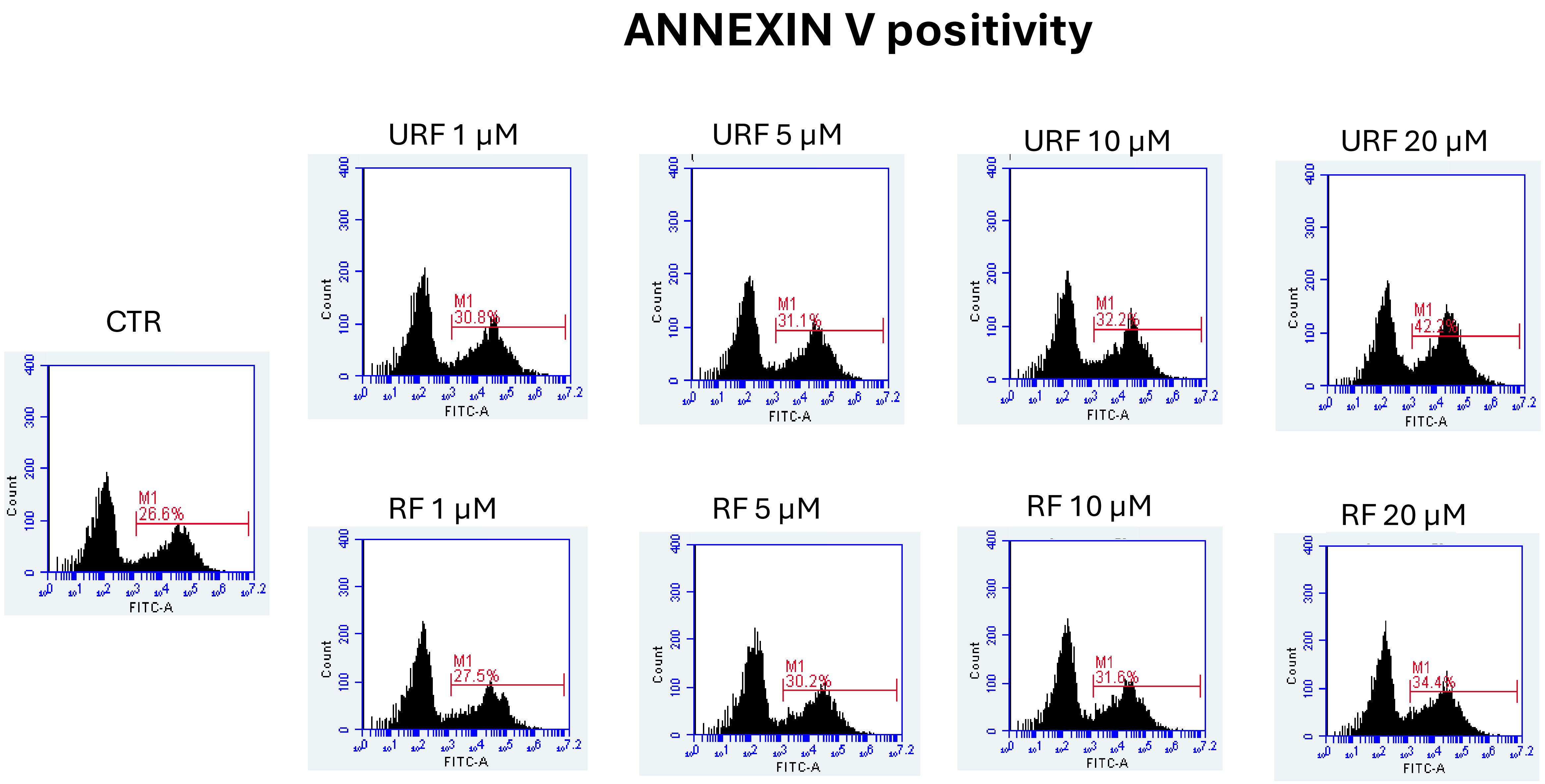

Figure S1. Original flow cytometry images showing the effect of different Annurca apple extracts on RBC eryptosis. The cells were treated with increasing concentrations of unripe flesh and ripe flesh.

ANNEXIN V positivity

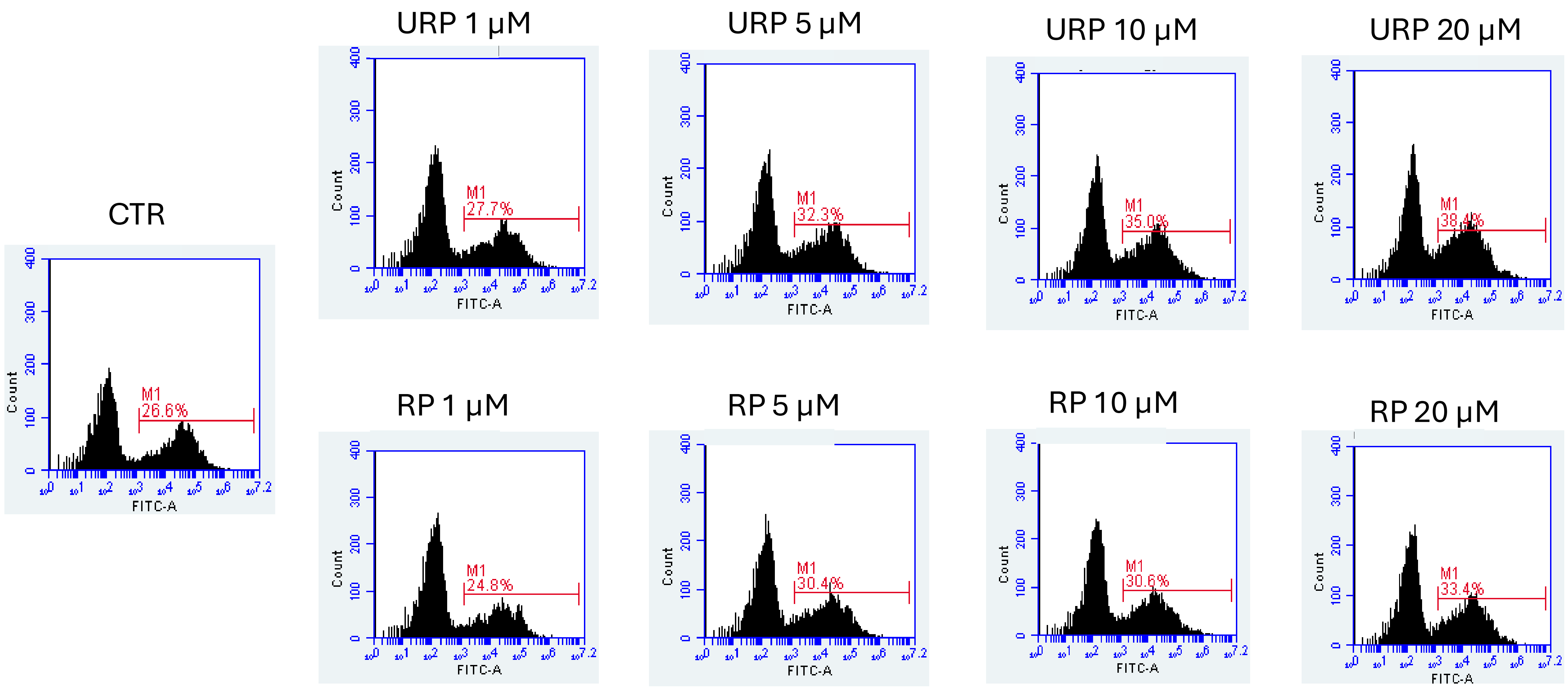

Figure S2. Original flow cytometry images showing the effect of different Annurca apple extracts on RBC eryptosis. The cells were treated with increasing concentrations of unripe peel and ripe peel.

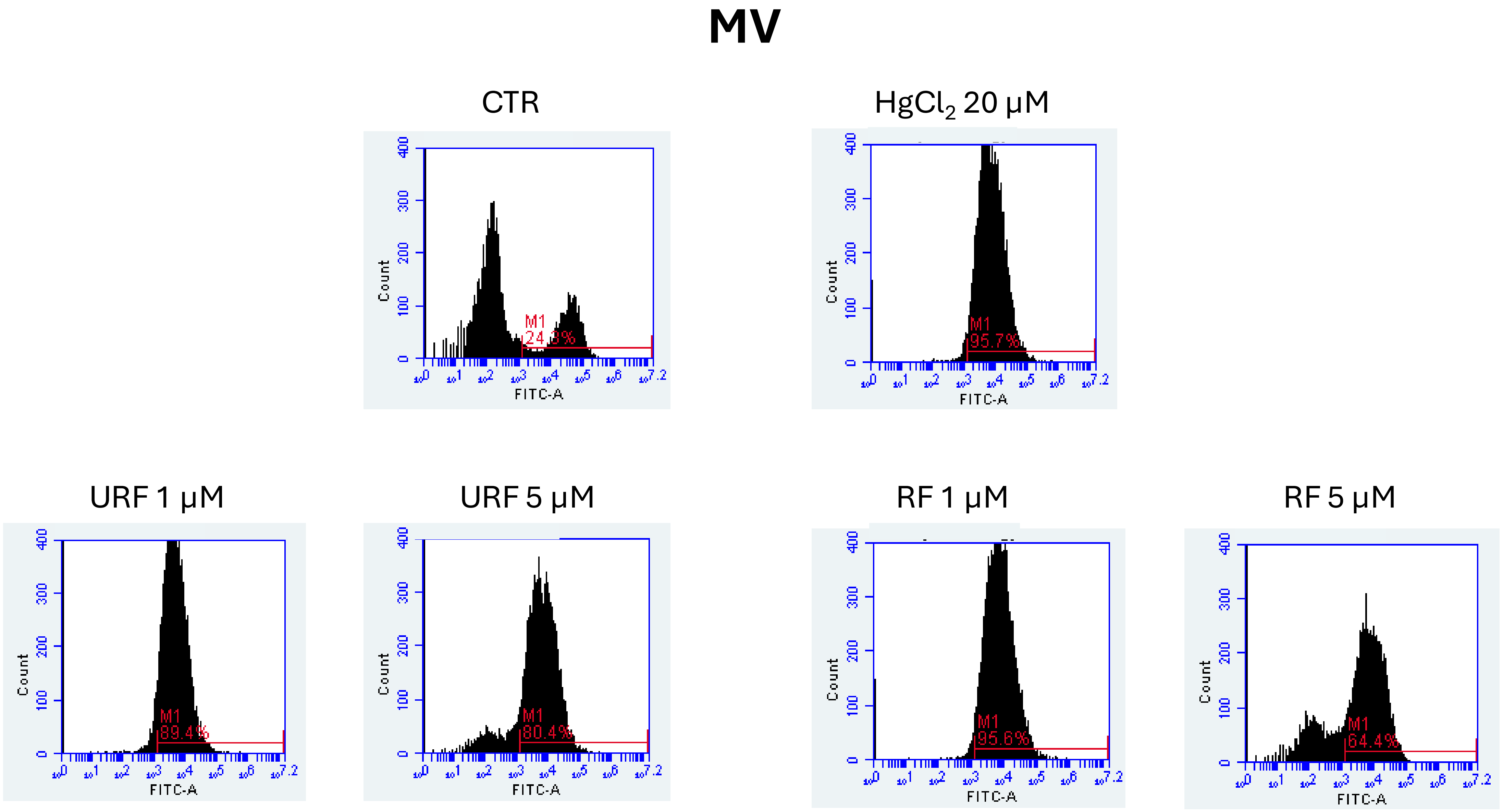

Figure S3. Original flow cytometry images showing the effect of different Annurca apple extracts on MV generation. The cells were treated with increasing concentrations of unripe flesh and ripe flesh.

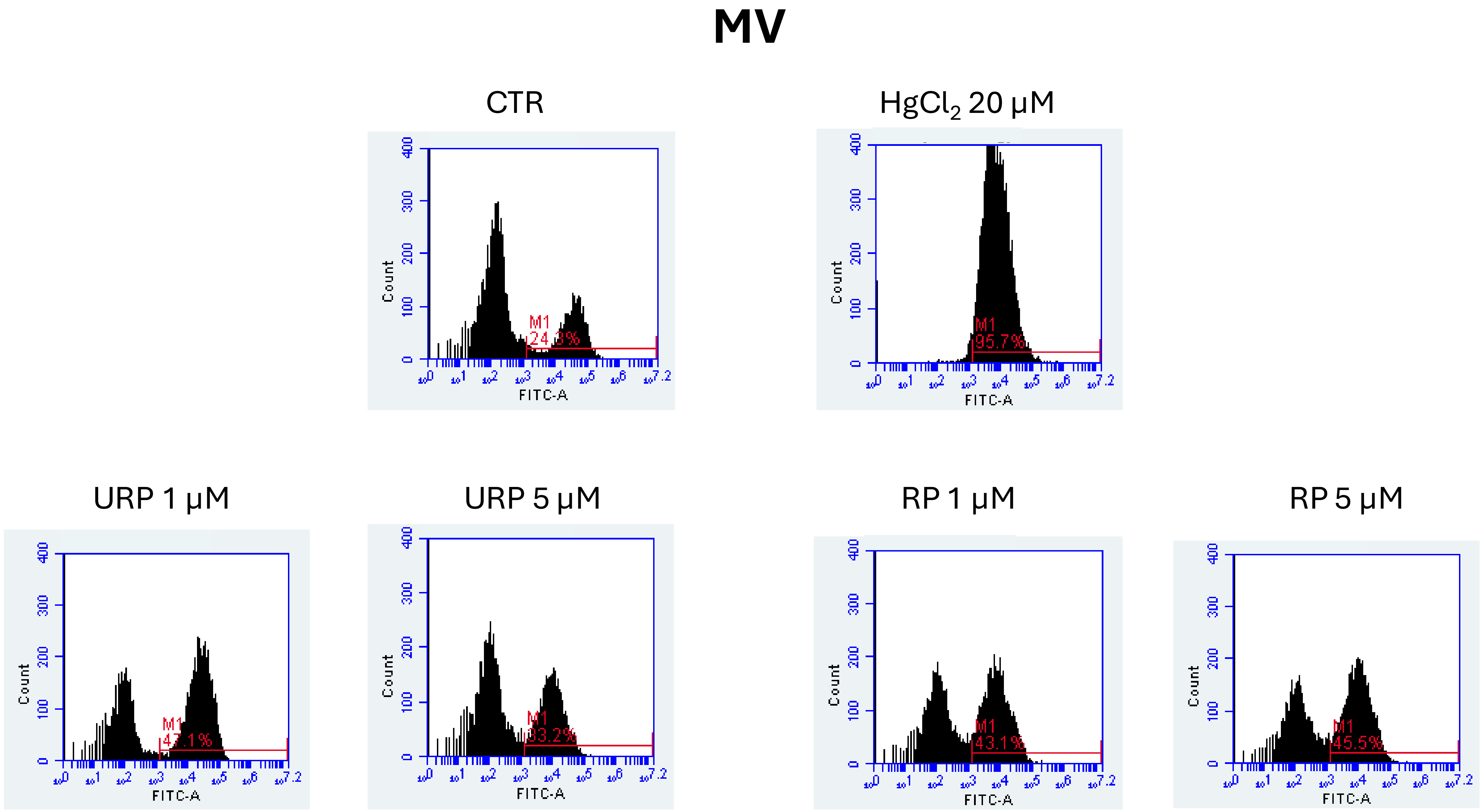

Figure S4. Original flow cytometry images showing the effect of different Annurca apple extracts on MV generation. The cells were treated with increasing concentrations of unripe peel and ripe peel.
